# The BioHub Knowledge Base: Ontology and Repository for Sustainable Biosourcing

**DOI:** 10.1186/s13326-016-0071-3

**Published:** 2016-06-01

**Authors:** Warren J. Read, George Demetriou, Goran Nenadic, Noel Ruddock, Robert Stevens, Jerry Winter

**Affiliations:** School of Computer Science, University of Manchester, Oxford Road, M13 9PL Manchester, UK; Unilever Research Port Sunlight, Bebington, Wirral, CH62 4ZD UK; Manchester Institute of Biotechnology, Princess St, Manchester M1 7DN, UK

**Keywords:** Ontology, Repository, Environmental sustainability, Chemistry

## Abstract

**Background:**

The motivation for the BioHub project is to create an Integrated Knowledge Management System (IKMS) that will enable chemists to source ingredients from bio-renewables, rather than from non-sustainable sources such as fossil oil and its derivatives.

**Method:**

The BioHubKB is the data repository of the IKMS; it employs Semantic Web technologies, especially OWL, to host data about chemical transformations, bio-renewable feedstocks, co-product streams and their chemical components. Access to this knowledge base is provided to other modules within the IKMS through a set of RESTful web services, driven by SPARQL queries to a Sesame back-end. The BioHubKB re-uses several bio-ontologies and bespoke extensions, primarily for chemical feedstocks and products, to form its knowledge organisation schema.

**Results:**

Parts of plants form feedstocks, while various processes generate co-product streams that contain certain chemicals. Both chemicals and transformations are associated with certain qualities, which the BioHubKB also attempts to capture. Of immediate commercial and industrial importance is to estimate the cost of particular sets of chemical transformations (leading to candidate surfactants) performed in sequence, and these costs too are captured. Data are sourced from companies’ internal knowledge and document stores, and from the publicly available literature. Both text analytics and manual curation play their part in populating the ontology. We describe the prototype IKMS, the BioHubKB and the services that it supports for the IKMS.

**Availability:**

The BioHubKB can be found via http://biohub.cs.manchester.ac.uk/ontology/biohub-kb.owl.

## Background

The aim of the BioHub project is to develop an Integrated Knowledge Management System (IKMS) that will enable chemists to source ingredients for chemical engineering processes from biorenewables rather than sourcing from non-renewable fossil feedstocks. An important component of the IKMS is the BioHub Knowledge Base (BioHubKB) which is an RDF store that uses an ontology written in the Web Ontology Language (OWL) [1] as a schema to organise knowledge about biorenewables; their component chemicals will be used by the IKMS to seed the exploration of possible chemical ingredients through application of cheminformatics algorithms [2].

One class of compounds of particular interest that drives the BioHub project are surfactants, which form the principal active ingredients in many personal care and household cleaning products. Thus the impetus of the BioHub project is to build an informatics infrastructure to support the development of surfactants and other chemicals, from sustainable agricultural feedstocks and co-product streams.

The use of such agricultural streams as chemical feedstocks is intended to obviate the need for sourcing chemicals from non-renewable fossil feedstocks, and to avoid the concomitant environmental costs associated with their extraction, refinement and use. The BioHub project seeks to facilitate the move from fossil fuel to biorenewable feedstocks. The process of exploring the sourcing of ingredients from biorenewables among the project partners is currently ad hoc, relying on reading public literature and proprietory documentation on chemical analyses, which are sometimes decades old. Gathering such knowledge into a central resource should facilitate the sourcing of novel ingredients. The project is a collaboration between Unilever and several other commercial and academic partner organisations. The commercial partners are motivated both to move to bio-renewables and to exploit more effectively their own materials streams.

The first use case in developing this prototype BioHub is to take co-product streams from the processing of sugar beet and identify the ingredients and transformations that can be chained together to generate good candidate surfactants. To make such a scenario work, the IKMS needs to enable chemists to describe the properties of a class of molecules they wish to derive from chemicals in bio-renewables. The IKMS will calculate a model of this class of chemicals by enumerating and selecting the possibilities with starting point chemicals [3] from knowledge in the BioHubKB about feedstocks and co-product streams and their component chemicals together with the chemical transformations in which they may participate.

The IKMS process takes chemicals, their properties and the transformations in which they may be involved, and supplies those data to a chemical enumeration routine. The BioHub’s hosted, web-based UI proposes sequences of candidate processes leading to candidate molecules, with both cost and chemical property prediction. In this paper we describe the BioHub’s knowledgebase, the BioHubKB, and its role in the IKMS.

The IKMS allows a chemist to specify the available co-product streams, the allowable transformations, and the specific desired chemical properties, before the chemical enumeration pipeline is run. A chemist might, for example, choose wool wax and rape oil as allowable source streams; s/he might go on to specify ozonolysis and ester hydrolysis as allowable transformations, with surfactancy as the desired property, e.g. a range of allowable surfactancy values as measured by critical micelle concentration (CMC). The input for the enumeration pipeline comes from the BioHubKB.

**Fig. 1:**
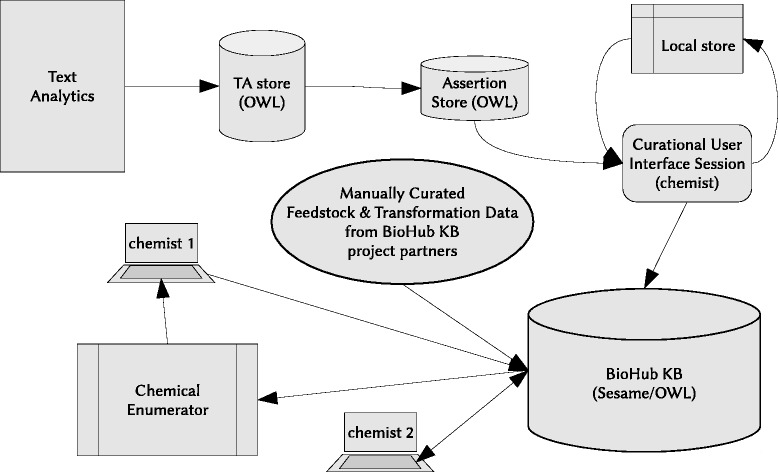
The BioHub IKMS

Figure 1 shows an overview of the IKMS. The components of the IKMS are: IKMS user interface: A web GUI that gives the chemist the ability to specify the application-specific requirement for a set of candidate molecules in suitable quantitative terms. Here the chemist may also select the allowable source streams of seed chemicals for the enumeration pipeline, as well as restricting the set of allowable transformations if necessary. IKMS enumeration pipeline: A set of routines that runs iteratively, which in each iteration computes the allowable transformations and products for all theoretically possible chemical reactions, based on a known set of substrates. These are then scored according to user-defined design criteria, Pareto-ranked and a subset chosen as inputs to the next iteration. In the first instance, the pipeline uses the known molecular constituents of co-product streams to seed the enumeration. Products of these reactions form the substrates for the next generation, and so on. BioHubKB: An RDF store with a schema formed from ontologies describing feedstocks, co-product streams, chemicals and transformations of those chemicals. A set of Web services offers methods to the enumeration pipeline to recover chemicals and the transformations in which they may participate.

Some example competencies [4] in the form of queries that the BioHubKB needs to support are:

1. Return a list of chemicals originating from a specified co-product stream, or set of co-product streams.
2. Return a list of chemicals that can undergo a single transformation, or at least one transformation from a supplied list.
3. Return a list of chemicals originating from among a supplied list of 1 or more co-product streams that are capable of undergoing 1 or more transformations among a separate supplied list.
4. Return the complete list of available chemical transformations.
5. Return the complete list of available co-product streams.
6. Return a list of co-product streams that are themselves a direct or downstream output of a specified upstream co-product stream (e.g. wool wax).

As well as being a repository for biorenewable feedstock data, the BioHub also contains curated details of industrially available chemical transformations. The BioHub application will provide chemists with query access to possible chemical products of chains of such transformations, starting from bio-sourced chemicals and thereafter using successive rounds of products as possible substrates for subsequent transformations. Queries specify desired chemical properties (e.g. relating to surfactancy) that are evaluated by one of several selectable computational chemical models; the model is also used as a generational filter to prevent the combinatorial explosion of chemical species.

## The BioHubKB

The BioHubKB is an ‘application ontology’ [5], where an ontology is created to fit a particular task model; in this case the production of surfactants, that addresses the competencies and scenario outlined above.

Where practicalities of scope, content and cognitive complexity permit, we have re-used extant, community based ontologies made by the Open Biomedical Ontologies consortium [6]. It re-uses (i) ChEBI [7] to describe its chemicals, and (ii) the Relations Ontology (RO) [8] for many of the relationships as well as some role hierarchy needed by the BioHubO. The Plant Ontology (PO) [9] is used to describe the parts of plants whence various feedstocks come. As well as plants, the IKMS will use animal based feedstocks meaning the BioHubO will need to be extended to animal species and the generic animal anatomy Uberon [10] wil be appropriately extended.

The design of the BioHub ontology was based on data sources that were made available to the developers by domain experts. Such sources included (i) corporate reports about chemical experiments and industrial processes (British Sugar), and (ii) internal spreadsheets/databases about specific chemicals and their potential sources and transformations (Unilever). The transformations for extracting streams and chemicals from sugar beet are well documented in the literature. Nevertheless, the developers paid particular attention to internal data used to describe the derivation of certain chemicals from sugar-beet for the development of the ontological structure of the sugar-beet representation. In addition, several key points in the ontology were discussed and clarified during consultation meetings with experts. Finally, external ontology resources, such as ChEBI, were used primarily to populate the BioHub ontology. In populating the BioHubKB, chemicals in input data needed to be mapped to chemicals in ChEBI and the representation of chemical transformations; this was accomplished using SMILES strings [11] present in ChEBI, plus SMILES and SMIRKS strings [12] from our in-house developed representations.

**Fig. 2:**
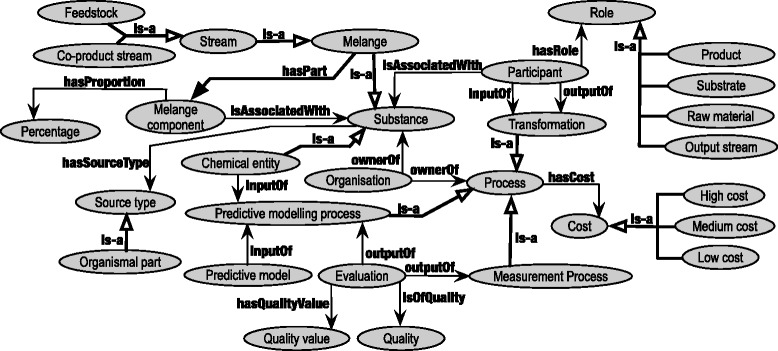
The domain general classes covering the scope of the BioHub ontology

The BioHubKB is authored in the Web Ontology Language (OWL) and classes etc. are denoted in this paper by the typeface Class name. Figure 2 shows the domain general classes of the BioHubKB, indicating its scope.

We have the class Substance that is a superclass for distinct chemical entities and mixtures, ‘slurries’ and ‘soups’ of chemical entities. We use ChEBI for distinct chemicals, but chemical engineering involves ‘soups’ and ‘slurries’ of mixtures of chemicals with various physical properties. The latter are covered by a class Melange, that is a superclass for continuous phase mixtures, plus soups/slurries which include components that co-exist in different phases. Formulation is a Melange of continuous phase. Stream is a Melange and is a superclass of Feedstock, Product stream and Coproduct stream.

Stream is a material stream that may be a completely unprocessed agricultural feedstock, or the output of a downstream process or series of processes (such as separation) applied to such a feedstock or its derivatives.

A Feedstock is an unprocessed, raw agricultural Stream such as Sugar beet feedstock (the freshly harvested plants themselves). Sugar beet, once harvested and entered into the processing is ‘playing the role’ of a feedstock; the Sugar beet feedstock is sourced from Whole sugar beet (within the Plant Ontology). ‘Feedstock’ may be a somewhat arbitrary role assigned to some raw materials. A sugar beet feedstock is itself separated (by cutting) into the sugar beet root and the sugar beet leaves, a ‘co-product’ for further processing.

**Fig. 3:**
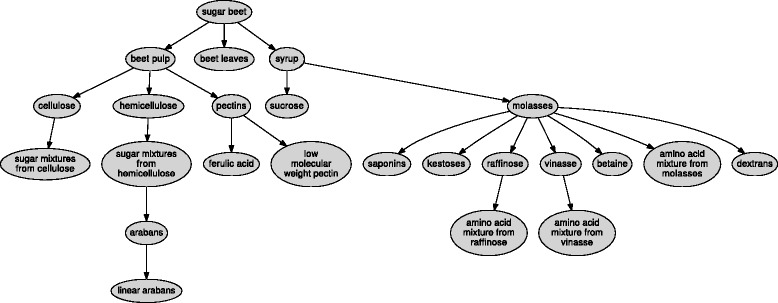
The entities and relationships that represent the sugar beet feedstock and some of its co-product streams. The arrows joining the streams represent Transformation processes such as Separation, Hydrolysis and Fermentation

A Coproduct stream is a stream of materials obtained as a co-product of processing an enterprise’s principal product(s). For example, Syrup is a Product stream derived from the Sugar beet feedstock and the Coproduct stream that is co-derived is Sugar beet pulp; this goes on for further refinement and produces (an)other Product stream(s) and Coproduct stream(s) until distinct chemical entities are produced. Each stream has its components of interest described (see below) down to chemical constituents and their proportions; for instance product stream Syrup has a large proportion of sucrose. Figure 3 shows how sugar beet and some of its co-product streams are represented as streams. The labels used for these streams are the ones used by the domain specialists with whom the BioHubO was created.

The BioHubKB classes below show sugar beet as a feedstock and some derivations into a product such as ‘syrup’ and co-product streams such as ‘sugar beet pulp’.



A SourceType is the type of source (typically extracted from a type of animal, vegetable, or mineral) whence a Substance derives. An OrganismalPart is an identifier assigned in the BioHubKB, to link a species ID, and an anatomical entity ID from Uberon or the Plant Ontology. For instance, Whole sugar beet is an organismal part whence Sugar beet feedstock comes (see above).

Transformation is a chemical Process used and/or made available industrially. For example, a transformation such as Separation will take Beet pulp and separate it into Cellulose, Hemicellulose and Pectin (subclasses of Coproduct stream), as well as Sugar mixtures (a Product stream). The class Separation can be further specialised to more precise ‘separations’, but the current representation is that required by our domain experts and the application needs.

Transformation also describes chemical processes such as Acetylation. Among a transformation’s annotations are SMIRKS strings that capture the reaction transforms. The SMIRKS annotations are used by the IKMS’s enumerator to evaluate which molecules (either from the source streams or the products from a previous iteration of the enumerator itself) constitute valid substrates for each transformation, based on their SMILES strings.

The class Participant expresses a ternary relation among a Substance, a Transformation and a Role; There is always an Input, an Output, or both, in relation to a Transformation. A MelangeComponent describes a part of a Melange, associated with a Substance (i.e. either a ChemicalEntity or another, nested Melange) and a proportion, expressed as a simple percentage. This enables BioHubKB to describe the yield of transformations.



A Role is a class imported from OBO RO, with various re-used subclasses from the same, e.g. Buffer, Solvent and Catalyst, in addition to other subclasses defined for the BioHubKB. These are chemical Substrate, which is an input of some Transformation, and chemical Product, which is an output from some Transformation. RawMaterial acts as input Stream to some Process. OutputStream acts as output Stream from Process.

A MeasurementProcess is a Process involving the quantitive measurement of some chemical property such as may relate to its surfactancy. A PredictiveModellingProcess is a process utilising a cheminformatics model to predict the attributes of a particular molecule. An Evaluation is a result generated by a Process, typically a MeasurementProcess or PredictiveModellingProcess.

An Organisation is an entity typically having an ownership relation to a Process or Substance.

The class Quality is used to represent a quality associated with an Evaluation, e.g. surface interfacial tension. QualityValue is a reifying class for associating unit and number with a Quality, for an Evaluation.

For a given transformation of a co-product stream a useful aspect for the IKMS is the percentage yield. We are interested in yield as a factor of the overall cost calculation associated with a Process, as applied to particular inputs and outputs. Yield is modelled as a Quality, with an associated QualityValue, which in turn has an associated unit (percentage in this case) and unitless number. Each QualityValue is associated with an Evaluation, which in the case of yield is the output of a MeasurementProcess (itself a subclass of Process).

A user of the IKMS will describe the class of chemicals to be generated through selection of physicochemical properties of that class. To take a familiar example, soaps with potential for use in laundry applications are a subclass of anionic surfactants which may be expected to exhibit characteristic traits, reflected in characteristic ranges in qualities like surface interfacial tension and CMC, as well as typically being easily derived from certain types of agricultural co-products. The modelling of physicochemical properties is difficult. The issues in doing so include the conditions in which a quality was measured, the devices used to do the measurement and the units for the measurement. There are several ontologies that could be used to capture some aspects of such measurements [13, 14], but pragmatic considerations led to a somewhat simplistic axiom pattern.

Actual feedstocks, products, co-products, etc. are represented as classes below the BioHubO general domain ontology to form the bulk of the BioHubKB. The content in the BioHubKB comes from spreadsheets supplied by our partners. We have a process to generate the BioHubKB using Tawny-OWL [15]. Spreadsheet worksheets are exported into comma-separated-variables (CSV) files, then read by Java code (via the OpenCSV library); the data then populate a Tawny-OWL script skeleton whose template format is defined by another Java library (FreeMarker). The populated template is then translated into OWL from Tawny-OWL.

The BioHub ontology was developed as described and then evaluated by project partners. We used a simple mechanism for evaluation whereby a diagram of the derivation of streams from feedstocks was presented along with a diagram of the gross structure of the ontology. These diagrams were associated with a simple survey [16]. The evaluation confirmed the overall representation in the ontology, but provided some useful extensions, in the form of missing steps, to the derivation paths. For example, instead of Ferulic acid being derived from Beet pulp, it was changed to reflect the true stepwise derivation of Ferulic acid, directly from Pectins. There were eight changes of this type. All proposed changes were made to the ontology.

### Querying the BioHubKB

The BioHubKB is typically queried automatically (via web services) by other components of the IKMS. One example is the IKMS user interface, which pre-populates its selection drop-downs (e.g. lists of streams and transformations) prior to any direct user interaction, based on the current content of the BioHubKB; another is the enumeration tool, which selects its initial “feed” molecules from those listed in the BioHubKB as belonging to the user-selected streams, and amenable to a set of user-selected transformations. However, the user (i.e. the chemist) him- or herself will also have the option to issue direct queries to the BioHubKB, such as, “Return a list of 3 chemicals found in the beet pulp stream, picked at random.”

## Discussion

The BioHubKB is a development of an application ontology that re-uses several OBO ontologies as well as being a *de novo* ontology that satisfies a series of competencies. It is used by a cheminformatics enumeration pipeline that generates a set of candidate chemicals based on a model specified by a user. If one of these candidate molecules is transformed from a chemical in the BioHubKB, then the BioHubKB contains the associated information about the feedstock whence the chemical came, such as the organisation that supplies that feedstock, its cost and the set of transformations the chemical underwent to yield the predicted chemical.

The current IKMS and its BioHubKB is a prototype to see if the approach is feasible. Hence there is much potential work to be done. The BioHubKB development has so far concentrated on sugar beet as a biorenewable; the other feedstocks are numerous—oil seed rape, as well as many other agricultural feedstocks, both plant and animal, and their co-products. Further development is also needed in the descriptions of chemical properties and their models used in the processing within the IKMS; incorporating some of the Ontology for Biomedical Investigations (OBI) [13] may help in this respect.

The goal of the IKMS that the BioHubKB supports is to facilitate the use of bio-renewable feedstocks in the chemical manufacturing process. This is necessarily a knowledge-driven process, a job for which Semantic Web technologies appear to be suited. That the bio-ontologies community has produced a range of ontologies that can be slotted into an artefact such as the BioHubKB, despite the scenario in which the IKMS is deployed not being traditional for the bio-ontology community, is a sign of the maturity and wider applicability of the community’s ontologies. This supports a model of a collection of reference ontologies that can be refactored and repurposed into application ontologies in a broad range of settings.

Ultimately, the BioHub has an auxiliary potential to act as a ‘marketplace’ for sustainable production, enabling chemists from diverse backgrounds and organisations to source bio-derived chemicals with multiple potential applications (including, but not limited to, surfactancy) from pre-existing industrial processing operations.
